# Tracking an Auto-Regressive Process with Limited Communication per Unit Time †

**DOI:** 10.3390/e23030347

**Published:** 2021-03-15

**Authors:** Rooji Jinan, Parimal Parag, Himanshu Tyagi

**Affiliations:** 1 Robert Bosch Centre for Cyber-Physical Systems, Indian Institute of Science, Bangalore 560012, India; 2 Department of ECE, Indian Institute of Science, Bangalore 560012, India; parimal@iisc.ac.in (P.P.); htyagi@iisc.ac.in (H.T.)

**Keywords:** auto-regressive process, quantization, successive update scheme

## Abstract

Samples from a high-dimensional first-order auto-regressive process generated by an independently and identically distributed random innovation sequence are observed by a sender which can communicate only finitely many bits per unit time to a receiver. The receiver seeks to form an estimate of the process value at every time instant in real-time. We consider a time-slotted communication model in a slow-sampling regime where multiple communication slots occur between two sampling instants. We propose a *successive update* scheme which uses communication between sampling instants to refine estimates of the latest sample and study the following question: Is it better to collect communication of multiple slots to send better refined estimates, making the receiver wait more for every refinement, or to be *fast but loose* and send new information in every communication opportunity? We show that the fast but loose successive update scheme with ideal spherical codes is universally optimal asymptotically for a large dimension. However, most practical quantization codes for fixed dimensions do not meet the ideal performance required for this optimality, and they typically will have a bias in the form of a fixed additive error. Interestingly, our analysis shows that the fast but loose scheme is not an optimal choice in the presence of such errors, and a judiciously chosen frequency of updates outperforms it.

## 1. Introduction

We consider the setting of real-time decision systems based on remotely sensed observations. In this setting, the decision maker needs to track the remote observations with high precision and in a timely manner. These are competing requirements, since high precision tracking will require larger number of bits to be communicated, resulting in larger transmission delay and increased staleness of information. Towards this larger goal, we study the following problem.

Consider a discrete time first-order auto-regressive (AR[1]) process $X_{t}\in R^{n}$, t≥0. A sensor draws a sample from this process, periodically once every *s* time-slots. In each of these time-slots, the sensor can send nR bits to a center. The center seeks to form an estimate ${\hat{X}}_{t}$ of $X_{t}$ at time *t*, with small mean square error (MSE). Specifically, we are interested in minimizing the time-averaged error $\sum_{t=1}^{T}E{\left\|X_{t}-{\hat{X}}_{t}\right\|}_{2}^{2}/T$ to enable timely and accurate tracking of $X_{t}$.

We propose and study a *successive update* scheme where the encoder computes the error in the estimate of the latest sample at the decoder and sends its quantized value to the decoder. The decoder adds this value to its previous estimate to update the estimate of the latest sample, and uses it to estimate the current value using a linear predictor. We instantiate this scheme with a general gain-shape quantizer for error-quantization.

Note that we can send this update several times between two sampling instances. In particular, our interest is in comparing a *fast but loose* scheme where an update is sent every slot against a scheme with slower update in every *p* communication slots. The latter allows the encoder to use more bits for the update, but the decoder will need to wait longer. We consider a class $X_{n}$ of discrete time AR[1] processes generated by an independently and identically distributed (*i.i.d*) random innovation sequence such that the fourth moment of the process is bounded. Within this class, we show that the fast but loose successive update scheme, used with an appropriately selected quantizer, is universally optimal for all possible distributions of the innovation sequence when the number of dimensions grows asymptotically large.

To show this optimality, we use a random construction for the quantizer, based on the spherical code given in [1,2]. Roughly speaking, this ideal quantizer *Q* yields

$$E{\left\|y-Q(y)\right\|}_{2}^{2}⩽{\left\|y\right\|}_{2}^{2}2^{-2R},$$

for every *n* dimensional vector *y* uniformly bounded. However, in practice, at finite *n*, such quantizers need not exist. Most practical vector quantizers have an extra additive error, i.e., the error bound takes the form

$$E{\left\|y-Q(y)\right\|}_{2}^{2}⩽{\left\|y\right\|}_{2}^{2}\theta +n\varepsilon^{2}$$

where θ and ϵ are quantizer parameters that varies with the choice of quantizer. We present our analysis for such general quantizers. Interestingly, for such a quantizer (which is all we have at a finite *n*), the optimal choice of *p* can differ from 1. Our analysis provides a theoretically sound guideline for choosing the frequency of updates 1/p for practical quantizers.

Our work relates to a large body of literature ranging from real-time compression to control and estimation over networks. The structure of real-time encoders for source coding has been studied in [3,4,5,6,7,8,9]. The general structure of real-time encoders for Markov sources is studied for communication over error-free channels in [3] and over noisy channels in [4,5]. The authors in [3] consider the communication delays similar to our setting. However, the delayed distortion criterion considered in [3] is different from the instantaneous distortion in our work. From these works, we see that optimal encoder output of a *k*th order Markov source at any instant would depend only on the *k* latest symbols and the present state of decoder memory. A similar structural result for the optimal encoders and decoders which are restricted to be causal is given in [6]. Furthermore, structural results in the context of optimal zero-delay coding of correlated sources are available in [7,8,9]. Some of these results can be extended to the case of finite delay decoding. However, as we need to track the process in real time, we have to produce an estimate for the latest sample at every instant even though the current information available at the decoder is stale and corresponds to past samples. This is quite different from the case of delayed decoding. Hence, the setup in all these works is different from the problem we consider and the results do not extend to our problem. Another related area which has been studied in literature is that of remote source coding of noisy sources [10,11,12] with an optimal encoder-decoder structure discussed in [11]. Further, [13] examines the optimal sampling strategy for remote reconstruction of a bandlimited signal from noisy versions of the source. Studies about remote reconstruction of a stationary process from its noisy/noiseless samples can be found in [14,15,16,17] as well. However, in our setting, the sampling is fixed and moreover each transmission in our system incurs a delay that depends on the encoding rate. Hence, our problem does not directly fit into any of these frameworks.

The problems of remote estimation under communication constraints of various kinds have been studied in [18,19,20,21,22,23]. This line of work proposes several Kalman-like recursive estimation algorithms and evaluates their performances. In a related thread, [24,25,26] study remote estimation under communication constraints and other related constraints using tools from dynamic programming and stochastic control. However, in all these works the role of channel delay is slightly different from that in our setting. Furthermore, the specific problem of choice of quantizer we consider has not been looked at. More recently, [27] studied remote estimation of Wiener process for channels with random delays and proves that the optimal sampling policy is a threshold based policy. This work, like some of the works cited above, assumes real-valued transmissions and does not take into account any quantization effects.

In more information theoretic settings, sequential coding for individual sequences under delay constraints was studied in [28,29,30,31]. Closer to our work, the causal (nonanticipatory) rate-distortion function for a stochastic process goes back to early works [32,33]. Recent works [34,35,36] consider the specific case of auto-regressive and Gauss–Markov processes and use the general formula in these early works to establish asymptotic optimality of simpler information structure for the encoders (optimal decoder structure is straightforward). Further, the system model in these works slightly differs from ours as the information transmission in our setting suffers a delay due to the channel rate constraint. We note that related formulations have been studied for simple settings of two or three iterations in [37,38] where interesting encoder structures for using previous communication for next sample as well emerge. Although some of these works propose specific optimal schemes as well, the key results in this line of research provide an expression for the rate-distortion function as an optimization problem, solving which will provide guidelines for a concrete scheme. In contrast, motivated by problems of estimation over an erasure channel, ref. [39] provides an asymptotically optimal scheme that roughly uses Gaussian codebooks to quantize the innovation errors between the encoder’s observation and the decoder’s estimation. Our work is closest to [39] and our encoding scheme shares similarities with the predictive Differential Pulse Code Modulation (DPCM) based scheme employed in [39]. However, our work differs in an important aspect from all the works mentioned in this research thread: we take transmission delays into account. In particular, in our formulation the estimation at the decoder happens in real time in spite of the communication delays. Note that the rate constraint in the channel causes a delay in the reception of information at the decoder. Nevertheless, the decoder must provide an estimate of the current state of the process at every time instant, and a longer codeword will result in a longer delay for the decoder to get complete information.

Nonetheless, our converse bound is derived using similar methods as [39]. Even the achievability part of our proof draws from [39], but there is a technical caveat. Note that after the first round of quantization, the error vector need not be Gaussian, and the analysis in [39] can only be applied after showing a closeness of the error vector distribution to Gaussian in the Wasserstein distance of order 2. While the original proof [39] overlooks this technical point, this gap can be filled using a recent result from [40] if spherical codes are used. However, we follow an alternative approach and show a direct analysis using vector quantizers.

In addition, there is a large body of work on control problems over rate-limited communication channels (cf. [41,42,43,44,45,46,47,48,49,50]). This line of work implicitly requires handling of communication delays in construction of estimators. However, the simple formulation we have seems to be missing and the results in this long line of work do not resolve the questions we raise.

Our main contributions in this paper are as follows: we present an encoder structure which we show to be optimal asymptotically in the dimension of the observation. Specifically, we propose to send successive updates that refines the estimates of the latest sample at the decoder. It is important to note that we quantize the estimation error at the decoder, instead of quantizing the innovation sequence formed at the encoder. Although the optimal MMSE decoder involves taking conditional expectation, we use a simple decoder which uses a simple linear structure. Yet, we show that this decoder is asymptotically optimal. Then, we instantiate this general scheme with spherical codes for quantizers to obtain a universal scheme. In particular, we consider general gain-shape quantizers and develop a framework to analyze their performance. One interesting result we present shows that the tradeoff between the accuracy and frequency of the updates must be carefully balanced based on the “bias” (additive error) in the quantizer used.

We present our problem formulation in the next section. Section 3 presents a discussion on the our achievability scheme followed by the main results in the subsequent section. Section 5 provides a detailed analysis of our scheme, which we further build-on in Section 6 to get our asymptotic achievability results. We prove our converse bound in Section 7 and conclude with a discussion on extensions of our result in the final section.

## 2. Problem Formulation

**Figure 1:**
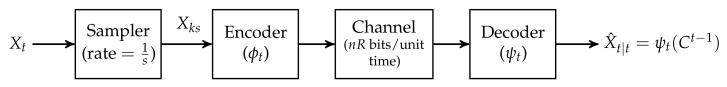
Communication model.

We begin by providing a formal description of our problem. Figure 1 illustrates the communication model adopted in this work. Different components of the model are presented in separate sections. Throughout the remainder of this paper, the set of real numbers is denoted by R, the set of positive reals is denoted by $R_{+}$, the *n*-dimensional Euclidean space is denoted by $R^{n}$ and the associated Euclidean norm by ${\left\|\cdot \right\|}_{2}$, the set of positive integers is denoted by N, the set of non-negative integers is denoted by $Z_{+}$, the set of continuous positive integers until *m* is denoted by [m]≜{1,⋯,m}, and an identity matrix of size n×n is denoted by $I_{n}$.

### 2.1. Observed Random Process and Its Sampling

For α∈(0,1), we consider a discrete time auto-regressive process of order 1 (AR[1] process) in $R^{n}$,

(1) $$X_{t}=\alpha X_{t-1}+\xi_{t},\;t⩾0,$$

where $\left(\xi_{t}\in R^{n},t⩾1\right)$ is an *i.i.d.* random sequence with zero mean and covariance matrix $\sigma^{2}\left(1-\alpha^{2}\right)I_{n}$. For simplicity, we assume that $X_{0}\in R^{n}$ is a zero mean random variable with covariance matrix $\sigma^{2}I_{n}$. This choice of covariance matrices for $\xi_{t}$ and $X_{0}$ ensures that the variance of $X_{t}\in R^{n}$ is $\sigma^{2}I_{n}$ for all t⩾0. Although this assumption makes the analysis more tractable, the dependence of the covariance matrix of $\xi_{t}$ on the process parameter α is not crucial for the analysis.

In addition, we assume that ${\left\|X_{t}\right\|}_{2}$ has a bounded fourth moment at all times t⩾0. Specifically, let κ>0 satisfy

$$\underset{k\in Z_{+}}{\sup}\frac{1}{n}\sqrt{E{\left\|X_{k}\right\|}_{2}^{4}}⩽\kappa .$$

It is clear that $X=(X_{t}\in R^{n},t⩾0)$ is a Markov process. We denote the set of processes *X* satisfying the assumptions above by $X_{n}$ and the class of all such processes for different choices of dimension *n* as X.

This discrete time process is subsampled periodically at *sampling frequency* 1/s, for some s∈N, to obtain samples $\left(X_{ks}\in R^{n},k⩾0\right)$.

### 2.2. Encoder Description

The sampled process $\left(X_{ks},k⩾0\right)$ is passed to an *encoder* which converts it to a bit stream. The encoder operates in *real-time* and sends nRs bits between any two sampling instants. Specifically, the encoder is given by a sequence of mappings ${\left(\phi_{t}\right)}_{t⩾0}$, where the mapping at any discrete time t=ks is denoted by

$$\phi_{t}:R^{n(k+1)}\to {\left\{0,1\right\}}^{nRs}.$$

The encoder output at time t=ks is denoted by the codeword $C_{ks}≜\phi_{k}\left(X_{0},X_{s},\cdots ,X_{ks}\right)$. We denote this codeword by an *s*-length sequence of binary strings $C_{ks}=\left(C_{ks,0},\cdots ,C_{ks,s-1}\right)$, where each term $C_{ks,i}$ takes values in ${\left\{0,1\right\}}^{nR}$. For time ks and 0⩽i⩽s−1, we can view the binary string $C_{ks,i}$ as the communication sent at time ks+i. We elaborate on the communication channel next.

### 2.3. Communication Channel

The output bit-stream of the encoder is sent to the receiver via an error-free communication channel. Specifically, we assume slotted transmission with synchronization where in each slot the transmitter sends nR bits of communication error-free. That is, we are allowed to send *R* bits per dimension, per time slot. Note that there is a delay of 1 time-unit (corresponding to one slot) in transmission of each nR bits. Therefore, the vector $C_{ks,i}$ of nR bits transmitted at time ks+i is received at time instant ks+i+1 for 0⩽i⩽s−1. Throughout we use the notation $I_{k}≜\left\{ks,\cdots ,(k+1)s-1\right\}$ and ${\tilde{I}}_{k}=I_{k}+1=\left\{ks+1,\cdots ,(k+1)s\right\}$, respectively, for the set of transmit and receive times for the strings $C_{ks,i}$, 0⩽i⩽s−1.

### 2.4. Decoder Description

We describe the operation of the receiver at time $t\in I_{k}$, for some k∈N, such that i=t−ks∈{0,⋯,s−1}. Upon receiving the codewords $C_{s},C_{2s},\ldots ,C_{(k-1)s}$ and the partial codeword $\left(C_{ks,0},\ldots ,C_{ks,i-1}\right)$ at time t=ks+i, the *decoder* estimates the current-state $X_{t}$ of the process using the estimator mapping

$$\psi_{t}:{\left\{0,1\right\}}^{nRt}\to R^{n}.$$

We denote the overall communication received by the decoder until time instant *t* by $C^{t-1}$. The time index t−1 in $C^{t-1}$ corresponds to the transmission time of the codewords, whereby the communication received till time *t* is denoted by $C^{t-1}$. Further, we denote by ${\hat{X}}_{t|t}$ the real-time causal estimate $\psi_{t}\left(C^{t-1}\right)$ of $X_{t}$ formed at the decoder at time *t*. Thus, the overall real-time causal estimation scheme is described by the mappings ${\left(\phi_{t},\psi_{t}\right)}_{t⩾0}$. It is important to note that the communication available to the decoder at time $t\in I_{k}$ can only depend on samples $X_{\ell }$ up to time ℓ⩽ks. As a convention, we assume that ${\hat{X}}_{0|0}=0$.

### 2.5. Performance Metrics

We call the encoder-decoder mapping sequence $\left(\phi ,\psi \right)={\left(\phi_{t},\psi_{t}\right)}_{t⩾0}$ a tracking code of rate *R* and sampling period *s*. The tracking error of our tracking code at time *t* for process *X* is measured by the mean squared error (MSE) per dimension given by

$$D_{t}\left(\phi ,\psi ,X\right)≜\frac{1}{n}E{\left\|X_{t}-{\hat{X}}_{t|t}\right\|}_{2}^{2}.$$

Our goal is to design (ϕ,ψ) with low average tracking error ${\bar{D}}_{T}\left(\phi ,\psi ,X\right)$ given by

$${\bar{D}}_{T}\left(\phi ,\psi ,X\right)≜\frac{1}{T}\sum_{t=0}^{T-1}D_{t}\left(\phi ,\psi ,X\right).$$

For technical reasons, we restrict to a finite time horizon setting. For the most part, the time horizon *T* will remain fixed and will be omitted from the notation. Instead of working with the mean-square error, a more convenient but equivalent parameterization for us will be that of accuracy, given by

$$\delta^{T}\left(\phi ,\psi ,X\right)=1-\frac{{\bar{D}}_{T}\left(\phi ,\psi ,X\right)}{\sigma^{2}}.$$

**Definition** **1** (Maxmin tracking accuracy). *The worst-case tracking accuracy for $X_{n}$ attained by a tracking code (ϕ,ψ) is given by*

$$\delta^{T}\left(\phi ,\psi ,X_{n}\right)=\underset{X\in X_{n}}{\inf}\delta^{T}\left(\phi ,\psi ,X\right).$$

*The maxmin tracking accuracy for $X_{n}$ at rate R and sampling period s is given by*

$$\delta_{n}^{T}\left(R,s,,X_{n}\right)=\underset{\left(\phi ,\psi \right)}{\sup}\delta^{T}\left(\phi ,\psi ,X_{n}\right),$$

*where the supremum is over all tracking codes (ϕ,ψ).*

The maxmin tracking accuracy $\delta_{n}^{T}\left(R,s,X_{n}\right)$ is the fundamental quantity of interest for us. Note that it is possible to provide a similar characterization of the performance in terms of MSE instead of accuracy and obtain the exact same result. Recall that *n* denotes the dimension of the observations in $X_{t}$ for $X\in X_{n}$ and *T* the time horizon. However, we will only characterize $\delta_{n}^{T}\left(R,s,X_{n}\right)$ asymptotically in *n* and *T*. Specifically, we define the asymptotic maxmin tracking accuracy as

$$\delta^{*}\left(R,s,X\right)=\underset{T\to \infty }{lim sup}\underset{n\to \infty }{lim sup}\delta_{n}^{T}\left(R,s,X_{n}\right).$$

We will provide a characterization of $\delta^{*}\left(R,s,X\right)$ and present a sequence of tracking codes that attains it. In fact, the tracking code we use is an instantiation of our successive update scheme, which we describe in the next section. It is important to note that our results may not hold if we switch the order of limits above: we need very large codeword lengths depending on a fixed finite time horizon *T*.

## 3. The Successive Update Scheme

In this section, we present our main contribution in this paper, namely the *Successive Update* tracking code. Before we describe the scheme completely, we present its different components. In every communication slot, the transmitter gets an opportunity to send nR bits. The transmitter may use it to send any information about a previously seen sample. There are various options for the encoder. For instance, it may use the current slot to send some information about a sample it had seen earlier. Or it may use all the slots between two sampling instants to send a quantized version of the latest sample. The information utilized by the decoder will be limited by the choice of structure of information transmission adopted at encoder. As the process we consider is Markov in nature, we choose to utilize all the transmission instants between two sampling instants to send information about the latest sample.

### 3.1. Encoder Structure: Refining the Error Successively

As mentioned earlier, the encoder and decoder that we employ in this work are similar to that in the DPCM scheme [39,51,52]. However, recall that we have multiple transmission opportunities between each sampling instance and the transmissions are delayed. This calls for the need for certain modifications which we explain in the following.

Let ${\hat{X}}_{ks|t}$ and ${\hat{X}}_{t|t}$ respectively denote the estimate for $X_{ks}$ and $X_{t}$ formed at the receiver at time *t*. Our encoder computes the error in the receiver estimate of the last process sample at each time instant *t*. Denoting the error at time $t\in I_{k}$ by $Y_{t}≜X_{ks}-{\hat{X}}_{ks|t}$, the encoder quantizes this error $Y_{t}$ and sends it as communication $C_{ks+1,i}$. At any time instant t=ks+i, we suppose that we estimate ${\hat{X}}_{t|t}$ from ${\hat{X}}_{ks|t}$. In particular, we suppose that ${\hat{X}}_{t|t}=\alpha^{i}{\hat{X}}_{ks|t}$. Simply speaking, our encoder computes and quantizes the error in the current estimate of the last sample at the decoder, and sends it to the decoder to enable the refinement of the estimate in the next time slot. While we have not been able to establish optimality of this encoder structure, our results will show its asymptotic optimality when the number of dimensions *n* goes to infinity.

Even within this structural simplification, a very interesting question remains. Since the process is sampled once in *s* time slots, we have, potentially, nRs bits to encode the latest sample. At any time $t\in {\tilde{I}}_{k}$, the receiver has access to $\left(C_{0},\cdots ,C_{(k-1)s}\right)$ and the partial codewords $\left(C_{ks,0},\cdots ,C_{ks,i-1}\right)$ for i=t−ks. A simple approach for the encoder is to use the complete codeword to express the latest sample and the decoder can ignore the partial codewords. This approach will result in slow but very accurate updates of the sample estimates. An alternative fast but loose approach will send nR quantizer codewords to refine estimates in every communication slot. Should we prefer fast but loose estimates or slow but accurate ones? Our results will shed light on this conundrum.

### 3.2. The Choice of Quantizers

In our description of the encoder structure above, we did not specify a key design choice, namely the choice of the quantizer. We will restrict ourselves to using the same quantizer to quantize the error in each round of communication. The precision of this quantizer will depend on whether we choose a fast but loose paradigm or a slow but accurate one. However, the overall structure will remain the same. Roughly speaking, we allow any gain-shape [53] quantizer which separately sends the quantized value of the gain ${\left\|y\right\|}_{2}$ and the shape $y/{\left\|y\right\|}_{2}$ for input *y*. Formally, we use the following abstraction.

**Definition** **2** ((θ,ε)-quantizer family). *Fix 0<M<∞. For 0⩽θ⩽1 and 0<ε, a quantizer Q with dynamic range M specified by a mapping $Q:R^{n}\to {\left\{0,1\right\}}^{nR}$ constitutes an nR bit*(θ,ε)-quantizer* if for every vector $y\in R^{n}$ such that ${\left\|y\right\|}_{2}^{2}⩽nM^{2}$, we have*

$$E{\left\|y-Q(y)\right\|}_{2}^{2}⩽{\left\|y\right\|}_{2}^{2}\theta \left(R\right)+n\varepsilon^{2}.$$

*Further, for a mapping $\theta :R_{+}\to \left[0,1\right]$, which is a decreasing function of rate R, a family of quantizers $Q=\{Q_{R}:R>0\}$ constitutes an*(θ,ε)-quantizer family* if for every R the quantizer $Q_{R}$ constitutes an nR bit (θ(R),ε)-quantizer.*

The expectation in the previous definition is taken with respect to the randomness in the quantizer, which is assumed to be shared between the encoder and the decoder for simplicity. For instance, in Lemma 5 we study a random codebook based construction for a (θ,ε)-quantizer and we assume that once a random codebook is picked by the encoder, it is made known to the decoder. The parameter *M*, termed the *dynamic range* of the quantizer, specifies the domain of the quantizer. When the input *y* does not satisfy ${\left\|y\right\|}_{2}⩽\sqrt{n}M$, the quantizer simply declares a failure, which we denote by ⊥. Our tracking code may use any such (θ,ε)-quantizer family. It is typical in any construction of a gain-shape quantizer to have a finite *M* and ε>0. Our analysis for finite *n* will apply to any such (θ,ε)-quantizer family and, in particular, will bring-out the role of the “bias” ε. However, when establishing our optimality result, we instantiate it using a random spherical code to get the desired performance.

### 3.3. Description of the Successive Update Scheme

All the conceptual components of our scheme are ready. Note that, we focus on updating the estimates of the latest observed sample $X_{ks}$ at the decoder. Our encoder successively updates the estimate of the latest sample at the decoder by quantizing and sending estimates for errors $Y_{t}$.

As discussed earlier, we must decide if we prefer a fast but loose approach or a slow but accurate approach for sending error estimates. To carefully examine this tradeoff, we opt for a more general scheme where the nRs bits available between two samples are divided into m=s/p subfragments of length nRp bits each. We use an nRp bit quantizer to refine error estimates for the latest sample $X_{ks}$ (obtained at time t=ks) every *p* slots and send the resulting quantizer codewords as partial tracking codewords $\left(C_{ks,jp},\ldots ,C_{ks,(j+1)p-1}\right)$, 0⩽j⩽m−1. Specifically, the *k*th codeword transmission interval $I_{k}$ is divided into *m* subfragments $I_{k,j}$, 1⩽j⩽m given by

$$I_{k,j}≜\left\{ks+jp,\cdots ,ks+(j+1)p-1\right\},\;0⩽j⩽m-1,$$

and $\left(C_{ks,jp},\ldots ,C_{ks,(j+1)p-1}\right)$ is transmitted in communication slots in $I_{k,j}$.

At time instant t=ks+jp+1 the decoder receives the *j*th subfragment $\left(C_{ks,t-ks},t\in I_{k,j}\right)$ of nRp bits and uses it to refine the estimate of the latest source sample $X_{ks}$. Note that the fast but loose and the slow but accurate regimes described above correspond to p=1 and p=s, respectively. In the middle of the interval $I_{k,j}$, the decoder ignores the partially received quantization code and retains the estimate ${\hat{X}}_{ks}$ of $X_{ks}$ formed at time ks+(j−1)p+1. It forms an estimate of the current state $X_{ks+i}$ by simply scaling ${\hat{X}}_{ks}$ by a factor of $\alpha^{i}$.

Finally, we impose one more additional simplification on the decoder structure. We simply update the estimate by adding to it the quantized value of the error. Thus, the decoder has a simple linear structure.

We can use any nRp bit quantizer (with an abuse of notation, we will use $Q_{p}$ instead of $Q_{Rp}$ to denote an nRp bit quantizer) $Q_{p}$ for the *n*-dimensional error vector, whereby this scheme can be easily implemented in practice if $Q_{p}$ can be implemented. For instance, we can use any standard gain-shape quantizer. The performance of most quantizers can be analyzed explicitly to render them a (θ,ε)-quantizer family for an appropriate *M* and function θ. Later, when analyzing the scheme, we will consider a $Q_{p}$ coming from a (θ,ε)-quantizer family and present a theoretically sound guideline for choosing *p*.

Recall that we denote the estimate of $X_{u}$ formed at the decoder at time t⩾u by ${\hat{X}}_{u|t}$. We start by initializing ${\hat{X}}_{0|0}=0$ and then proceed using the encoder and the decoder algorithms outlined above. Note that our quantizer $Q_{p}$ may declare failure symbol ⊥, in which case the decoder must still yield a nominal estimate. We will simply declare the estimate as (In analysis, we account for all these events as error. Only the probability of failure will determine the contribution of this part to the MSE since the process is mean-square bounded.) 0 once a failure happens.

We give a formal description of our encoder and decoder algorithms below.

**The encoder.**

1 Initialize k=0, j=0, ${\hat{X}}_{0|0}=0$.
2 At time t=ks+jp, use the decoder algorithm (to be described below) to form the estimate ${\hat{X}}_{ks|t}$ and compute the error
  (2) $$Y_{k,j}≜X_{ks}-{\hat{X}}_{ks|t},$$
  where we use the latest sample $X_{ks}$ available at time t=ks+jp.
3 Quantize $Y_{k,j}$ to nRp bit as $Q_{p}\left(Y_{k,j}\right)$.
4 If quantize failure occurs and $Q_{p}\left(Y_{k,j}\right)=⊥$, send ⊥ to the receiver and terminate the encoder.
5 Else, send a binary representation of $Q_{p}\left(Y_{k,j}\right)$ as the communication $\left(C_{ks,0},...,C_{ks,p-1}\right)$ to the receiver over the next *p* communication slots (For simplicity, we do not account for the extra message symbol needed for sending ⊥.).
6 If j<m−1, increase *j* by 1; else set j=0 and increase *k* by 1. Go to Step 2.

**The decoder.**

1 Initialize k=0, j=0, ${\hat{X}}_{0|0}=0$.
2 At time t=ks+jp, if encoding failure has not occurred until time *t*, compute
  $${\hat{X}}_{ks|ks+jp}={\hat{X}}_{ks|ks+(j-1)p}+Q_{p}\left(Y_{k,j-1}\right),$$
  and output ${\hat{X}}_{t|t}=\alpha^{t-ks}{\hat{X}}_{ks|t}$.
3 Else, if encoding failure has occurred and the ⊥ symbol is received declare ${\hat{X}}_{s|t}=0$ for all subsequent time instants s⩾t.
4 At time t=ks+jp+i, for i∈[p−1], output (We ignore the partial quantizer codewords received as $\left(C_{ks,jp+1},C_{ks,jp+2},\cdots ,C_{ks,jp+i-1}\right)$ till time *t*.) ${\hat{X}}_{t|t}=\alpha^{t-ks}{\hat{X}}_{ks|ks+jp}$.
5 If j<m−1, increase *j* by 1; else set j=0 and increase *k* by 1. Go to Step 2.

Note that the decoder has a simple structure and the principal component of the encoder is the quantizer. Therefore, the complexity of the proposed scheme is dominated by the complexity of the quantization operation and varies with respect to the quantizer chosen.

## 4. Main Results

We present results in two categories. First, we provide an explicit formula for the asymptotic maxmin tracking accuracy $\delta^{*}\left(R,s,X\right)$. Next, we present a theoretically-founded guideline for selecting a good *p* for the successive update scheme with a (θ,ε)-quantizer family. Interestingly, the optimal choice may differ from the asymptotically optimal choice of p=1.

### 4.1. Characterization of the Maxmin Tracking Accuracy

To describe our result, we define the functions $\delta_{0}:R_{+}\to \left[0,1\right]$ and $g:R_{+}\to \left[0,1\right]$ as

(3) $$\begin{array}{cc} \delta_{0}\left(R\right) & ≜\frac{\alpha^{2}\left(1-2^{-2R}\right)}{\left(1-\alpha^{2}2^{-2R}\right)},\;\mathrm{for}\;\mathrm{all}\;R>0;\\ g(s) & ≜\frac{\left(1-\alpha^{2s}\right)}{s(1-\alpha^{2})},\;\mathrm{for}\;\mathrm{all}\;s>0. \end{array}$$

Note that g(s) is a decreasing function of *s* with g(1)=1. The result below shows that, for an appropriate choice of the quantizer, our successive update scheme with p=1 (the fast but loose version) achieves an accuracy of $\delta_{0}\left(R\right)g\left(s\right)$ asymptotically, universally for all processes in X.

**Theorem** **1** (Lower bound for maxmin tracking accuracy: The achievability). *For R>0 and s∈N, the asymptotic maxmin tacking accuracy is bounded below as*

$$\delta^{*}\left(R,s,X\right)⩾\delta_{0}\left(R\right)g\left(s\right).$$

*Furthermore, this bound can be obtained by a successive update scheme with p=1 and appropriately chosen quantizer $Q_{p}$.*

We provide a proof in Section 6. Note that while we assume that the per dimension fourth moment of the processes in X is bounded, the asymptotic result above does not depend on that bound. Interestingly, the performance characterized above is the best possible.

**Theorem** **2** (Upper bound for maxmin tracking accuracy: The converse). *For R>0 and s∈N, the asymptotic maxmin tacking accuracy is bounded above as*

$$\delta^{*}\left(R,s,X\right)⩽\delta_{0}\left(R\right)g\left(s\right).$$

*Furthermore, the upper bound is obtained by considering a Gauss–Markov process.*

We provide a proof in Section 7. Thus, $\delta^{*}\left(R,s,X\right)=\delta_{0}\left(R\right)g\left(s\right)$ with the *fast but loose* successive update scheme being universally (asymptotically) optimal and the Gauss–Markov process being the most difficult process to track. Clearly, the best possible choice of sampling period is s=1 and the highest possible accuracy at rate *R* is $\delta_{0}\left(R\right)$, whereby we cannot hope for an accuracy exceeding $\delta_{0}\left(R\right)$.

To provide an alternative view of the result, suppose that we fix the achievable tracking accuracy to be $\delta ⩽\delta_{0}\left(R\right)$ at rate *R*. Then, the above result says that $g\left(s\right)⩾(\delta /\delta_{0}\left(R\right))$ where g(s) is a decreasing function of *s*. Therefore, this result can be interpreted as saying that we cannot subsample at a frequency less than $1/\lfloor g^{-1}\left(\delta /\delta_{0}\left(R\right)\right)\rfloor$ for attaining a tracking accuracy $\delta ⩽\delta_{0}\left(R\right)$.

### 4.2. Guidelines for Choosing a Good *p*

The proof of Theorem 1 entails the analysis of the successive update scheme for p=1. In fact, we can analyze this scheme for any p∈N and for any (θ,ε)-quantizer family; we term this tracking code the *p-successive update* (*p*-SU) scheme. This analysis can provide a simple guideline for the optimal choice of *p* depending on the performance of the quantizer.

However, there are some technical caveats. A quantizer family will operate only as long as the input *y* satisfies ${\left\|y\right\|}_{2}⩽M$. If a *y* outside this range is observed, the quantizer will declare ⊥ and the tracking code encoder, in turn, will declare a failure. We denote by τ the stopping time at which encoder failure occurs for the first time, i.e.,

$$\tau ≜\min\{ks+jp:Q_{p}\left(Y_{k,j}\right)=⊥,0⩽k,0⩽j⩽m-1\}.$$

Further, denote by $A_{t}$ the event that failure does not occur until time *t*, i.e.,

$$A_{t}≜\left\{\tau >t\right\}.$$

We characterize the performance of a *p*-SU in terms of the probability of encoder failure in a finite time horizon *T*.

**Theorem** **3** (Performance of *p*-SU). *For fixed θ,ε,β∈[0,1], consider the p-SU scheme with an nRp bit (θ,ε)-quantizer $Q_{p}$, and denote the corresponding tracking code by $\left(\phi_{p},\psi_{p}\right)$. Suppose that for a time horizon T∈N, the tracking code $\left(\phi_{p},\psi_{p}\right)$ satisfies $P\left(\tau ⩽T\right)⩽\beta$. Then,*

$$\underset{X\in X_{n}}{\sup}{\bar{D}}^{T}\left(\phi_{p},\psi_{p},X\right)⩽B_{T}\left(\theta ,\varepsilon ,\beta \right),$$

*where $B_{T}\left(\theta ,\varepsilon ,\beta \right)$ satisfies*

$$\begin{array}{c} \underset{T\to \infty }{lim sup}B_{T}\left(\theta ,\varepsilon ,\beta \right)⩽\sigma^{2}\left[1-\frac{g\left(s\right)\;\alpha^{2p}}{1-\alpha^{2p}\;\theta }\left(1-\frac{\varepsilon^{2}}{\sigma^{2}}-\theta \right)\right]+\frac{\kappa \beta g(s)}{\left(1-\alpha^{2s}\right)}\left(1-\alpha^{2(s+p)}\frac{\left(1-\theta \right)}{1-\alpha^{2p}\theta }\right). \end{array}$$

We provide the proof of this theorem later in Section 5. We remark that β can be made small by choosing *M* to be large for a quantizer family. Furthermore, the inequality in the upper bound for the MSE in the previous result (barring the dependence on β) comes from the inequality in the definition of a (θ,ε)-quantizer, rendering it a good proxy for the performance of the quantizer. The interesting regime is that of very small β where the encoder failure does not occur during the time horizon of operation. If we ignore the dependence on β, the accuracy of the *p*-SU does not depend either on *s* or on the bound for the fourth moment κ. Motivated by these insights, we define the *accuracy-speed* curve of a quantizer family as follows.

**Definition** **3** (The accuracy-speed curve). *For α∈[0,1], $\sigma^{2}$, and R>0, the *accuracy-speed* curve for a (θ,ε)-quantizer family Q is given by*

$$\Gamma_{Q}\left(p\right)=\frac{\alpha^{2p}}{1-\alpha^{2p}\;\theta \left(Rp\right)}\left(1-\frac{\varepsilon^{2}}{\sigma^{2}}-\theta \left(Rp\right)\right),\;p>0.$$

By Theorem 3, it is easy to see that the accuracy (precisely the upper bound on the accuracy) of a *p*-SU scheme is better when $\Gamma_{Q}\left(p\right)$ is larger. Thus, a good choice of *p* for a given quantizer family *Q* is the one that maximizes $\Gamma_{Q}\left(p\right)$ for 1⩽p⩽s.

We conclude by providing accuracy-speed curves for some illustrative examples. To build some heuristics, note that a uniform quantization of [−M,M] has θ(R)=0 and $\varepsilon =M2^{-R}$. For a gain-shape quantizer, we express a vector $y={\left\|y\right\|}_{2}y_{s}$ where the shape vector $y_{s}$ has ${\left\|y_{s}\right\|}_{2}=1$. An ideal shape quantizer (which only can be shown to exist asymptotically) using *R* bits per dimension will satisfy $E{\left\|\hat{y_{s}}-y_{s}\right\|}_{2}^{2}⩽2^{-2R}$, similar to the scalar uniform quantizer. In one of the examples below, we consider gain-shape quantizers with such an ideal shape quantizer.

**Example** **1.** *We begin by considering an ideal quantizer family with $\theta \left(R\right)=2^{-2R}$ and ε=0. In our asymptotic analysis, we will show roughly that such a quantizer with very small ε exists. For this ideal case, for R>0, the accuracy-speed curve is given by*

$$\Gamma_{Q}\left(p\right)=\frac{\alpha^{2p}-\alpha^{2p}\;\theta \left(Rp\right)}{1-\alpha^{2p}\;\theta \left(Rp\right)}=1-\frac{1-\alpha^{2p}}{1-\alpha^{2p}2^{-Rp}}.$$

*It can be seen that $\Gamma_{Q}\left(p\right)$ is decreasing in p whereby the optimal choice of p that maximized $\Gamma_{Q}\left(p\right)$ over p∈[s] is p=1. Heuristically, this justifies why asymptotically the fast but loose successive update scheme is optimal.*

**Example** **2** (Uniform scalar quantization). *In this example, we consider a coordinate-wise uniform quantizer. Since we seek quantizers for inputs $y\in R^{n}$ such that ${\left\|y\right\|}_{2}⩽M\sqrt{n}$, we can only use uniform quantizer of $\left[-M\sqrt{n},M\sqrt{n}\right]$ for each coordinate. For this quantizer, we have θ=0 and $\varepsilon^{2}=nM^{2}2^{-2R}$, whereby the accuracy-speed curve is given by $\Gamma_{Q}\left(p\right)=\alpha^{2p}\left(1-nM^{2}2^{-2R}/\sigma^{2}\right)$. Thus, once again, the optimal choice of p that maximizes accuracy is p=1.*

**Example** **3** (Gain-shape quantizer). *Consider the quantization of a vector $y=ay_{s}$ where $a={\left\|y\right\|}_{2}$. The vector y is quantized by a gain-shape quantizer which quantizes the norm and shape of the vector separately to give $Q\left(y\right)=\hat{a}{\hat{y}}_{s}$. We use a uniform quantizer within a fixed range $\left[0,M\sqrt{n}\right]$ in order to quantize the norm a to $\hat{a}$, where an ideal shape quantizer is employed in quantizing the shape vector $y_{s}$. Namely, we assume $E{\left\|y_{s}-{\hat{y}}_{s}\right\|}_{2}^{2}⩽2^{-2R}$ and $\|{\hat{y}}_{s}\|⩽1$. Suppose that we allot ℓ bits out of the total budget of nR bits for norm quantization and the rest for shape quantization. Then, we see that*

$$E{\left\|y-Q(y)\right\|}_{2}^{2}⩽2a^{2}2^{-2(R-\ell /n)}+nM^{2}2^{-2\ell -1},$$

*whereby $\theta \left(R\right)=2^{-2(R-\ell /n)+1}$ and $\epsilon^{2}=M^{2}2^{-2\ell -1}$. Thus, the accuracy-speed curve is given by*

$$\begin{array}{c} \Gamma_{Q}\left(p\right)=\frac{\alpha^{2p}}{1-2\alpha^{2p}2^{-2(Rp-\ell /n)}}\;\left(1-\frac{2M^{2}2^{-2\ell -1}}{\sigma^{2}}-2^{-2(Rp-\ell /n)+1}\right). \end{array}$$

*Note that the optimal choice of p in this case depends on the choice of M.*

We illustrated application of our analysis for idealized quantizers, but it can be used to analyze even very practical quantizers, such as the recently proposed almost optimal quantizer in [54].

## 5. Analysis of the Successive Update Scheme

From the discussion in Section 3, we observe that the successive update scheme is designed to refine the estimate of $X_{ks}$ in each interval ${\tilde{I}}_{k}$. This fact helps us in establishing a recursive relation for $D_{t}\left(\phi_{p},\psi_{p},X\right)$, $t\in {\tilde{I}}_{k}$ in terms of $D_{ks}\left(\phi_{p},\psi_{p},X\right)$ which is provided next.

**Lemma** **1.** *For a time instant t=ks+jp+i,0⩽j⩽m−1,0⩽i⩽p−1 and k⩾0, let $\left(\phi_{p},\psi_{p}\right)$ denote the tracking code of a p-SU scheme employing an nRp bit (θ,ϵ)-quantizer. Assume that $P\left(A_{t}^{c}\right)⩽\beta^{2}$. Then, we have*

$$\begin{array}{c} D_{t}\left(\phi_{p},\psi_{p},X\right)⩽\alpha^{2(t-ks)}\theta^{j}D_{ks}\left(\phi_{p},\psi_{p},X\right)+\sigma^{2}\left(1-\alpha^{2(t-ks)}\right)+\frac{\alpha^{2(t-ks)}\left(1-\theta^{j}\right)\varepsilon^{2}}{\left(1-\theta \right)}+\alpha^{2(t-ks)}\kappa \beta . \end{array}$$

**Proof.**  From the evolution of the AR[1] process defined in (1), we see that $X_{t}=\alpha^{t-ks}X_{ks}+\sum_{u=ks+1}^{t}\alpha^{t-u}\xi_{u}$. Further for the *p*-SU scheme, we know that ${\hat{X}}_{t|t}=\alpha^{t-ks}{\hat{X}}_{ks|ks+jp}$ at each instant t=ks+jp+i. Therefore, we have

$$X_{t}-{\hat{X}}_{t|t}=\alpha^{t-ks}\left(X_{ks}-{\hat{X}}_{ks|ks+jp}\right)+\sum_{u=ks+1}^{t}\alpha^{t-u}\xi_{u}.$$

Since the estimate ${\hat{X}}_{ks|ks+jp}$ is a function of samples $\left(X_{0},\cdots ,X_{ks}\right)$, and the sequence $\left(\xi_{u},u⩾ks\right)$ is independent of the past, we obtain the per dimension MSE as

$$\begin{array}{c} D_{t}\left(\phi_{p},\psi_{p},X\right)=\frac{\alpha^{2(t-ks)}}{n}E{\left\|X_{ks}-{\hat{X}}_{ks|ks+jp}\right\|}_{2}^{2}+\sigma^{2}\left(1-\alpha^{2(t-ks)}\right). \end{array}$$

Further, we divide the error into two terms based on occurrence of the failure event as follows:

(4) $$\begin{array}{c} D_{t}\left(\phi_{p},\psi_{p},X\right)=\frac{\alpha^{2(t-ks)}}{n}\left[E\left[{\left\|X_{ks}-{\hat{X}}_{ks|ks+jp}\right\|}_{2}^{2}{𝟙}_{A_{t}}\right]+E\left[{\left\|X_{ks}-{\hat{X}}_{ks|ks+jp}\right\|}_{2}^{2}{𝟙}_{A_{t}^{c}}\right]\right]\\ +\sigma^{2}\left(1-\alpha^{2(t-ks)}\right). \end{array}$$

Recall that at each instant t=ks+jp, we refine the estimate ${\hat{X}}_{ks|ks+(j-1)p}$ of $X_{ks}$ to ${\hat{X}}_{ks|ks+jp}=\left({\hat{X}}_{ks|ks+(j-1)p}+Q_{p}\left(Y_{k,j-1}\right)\right){𝟙}_{A_{t}}$. Upon substituting this expression for ${\hat{X}}_{ks|ks+jp}$, we obtain

$$\begin{array}{cc} E[{\left\|X_{ks}-{\hat{X}}_{ks|ks+jp}\right\|}_{2}^{2}{𝟙}_{A_{t}}] & =E[{\left\|Y_{k,j-1}-Q_{p}\left(Y_{k,j-1}\right)\right\|}^{2}{𝟙}_{A_{t}}]\\ & ⩽\theta E\left[{\left\|X_{ks}-{\hat{X}}_{ks|ks+(j-1)p}\right\|}_{2}^{2}{𝟙}_{A_{t}}\right]+n\varepsilon^{2}, \end{array}$$

where the identity uses the definition of error $Y_{k,j-1}$ given in (2) and the inequality holds since $Q_{p}$ is a (θ,ε)-quantizer. Repeating the previous step recursively, we get

$$\begin{array}{cc} \frac{1}{n}E\left[{\left\|X_{ks}-{\hat{X}}_{ks|ks+jp}\right\|}_{2}^{2}{𝟙}_{A_{t}}\right] & ⩽\theta^{j}\cdot \frac{1}{n}E\left[{\left\|X_{ks}-{\hat{X}}_{ks|ks}\right\|}_{2}^{2}{𝟙}_{A_{t}}\right]+\frac{1-\theta^{j}}{1-\theta }\cdot \varepsilon^{2}\\ & ⩽\theta^{j}\cdot \frac{1}{n}E{\left\|X_{ks}-{\hat{X}}_{ks|ks}\right\|}_{2}^{2}+\frac{1-\theta^{j}}{1-\theta }\cdot \varepsilon^{2}, \end{array}$$

which is the same as

$$\begin{array}{c} \frac{1}{n}E\left[{\left\|X_{ks}-{\hat{X}}_{ks|ks+jp}\right\|}_{2}^{2}{𝟙}_{A_{t}}\right]⩽\theta^{j}\cdot D_{ks}\left(\phi_{p},\psi_{p},X\right)+\frac{1-\theta^{j}}{1-\theta }\cdot \varepsilon^{2}. \end{array}$$

Moving to the error term $E[{\left\|X_{ks}-{\hat{X}}_{ks|ks+jp}\right\|}_{2}^{2}{𝟙}_{A_{t}^{c}}]$ when encoder failure occurs, recall that the decoder sets the estimate to 0 in the event of an encoder failure. Thus, using the Cauchy–Schwarz inequality, we get

$$\begin{array}{cc} \frac{1}{n}E\left[{\left\|X_{ks}-{\hat{X}}_{ks|ks+jp}\right\|}_{2}^{2}{𝟙}_{A_{t}^{c}}\right] & =\frac{1}{n}E\left[{\left\|X_{ks}\right\|}^{2}{𝟙}_{A_{t}^{c}}\right]\\ & ⩽\frac{1}{n}\sqrt{E\left[{\left\|X_{ks}\right\|}^{4}\right]P\left(A_{t}^{c}\right)}\\ & ⩽\kappa \beta . \end{array}$$

Substituting the two bounds above in (4), we get the result. □

The following recursive bound can be obtained using almost the same proof as that of Lemma 1; we omit the details.

**Lemma** **2.** *Let $\left(\phi_{p},\psi_{p}\right)$ denote the tracking code of a p-SU scheme employing an nRp bit (θ,ϵ)-quantizer. Assume that $P\left(A_{t}^{c}\right)⩽\beta^{2}$. Then, we have*

$$\begin{array}{c} D_{ks}\left(\phi_{p},\psi_{p},X_{n}\right)⩽\alpha^{2s}\theta^{m}D_{(k-1)s}\left(\phi_{p},\psi_{p},X_{n}\right)+\sigma^{2}\left(1-\alpha^{2s}\right)+\alpha^{2s}\varepsilon^{2}\frac{\left(1-\theta^{m}\right)}{\left(1-\theta \right)}+\alpha^{2s}\kappa \beta . \end{array}$$

We also need the following technical observation.

**Lemma** **3.** *For a sequence $\left(X_{k}\in R:k\in Z_{+}\right)$ that satisfies sequence of upper bounds*

$$X_{k}⩽aX_{k-1}+b,\;\forall \;k\in Z_{+},$$

*with constants a,b∈R such that b is finite and a∈(−1,1), we have*

$$\lim_{K\to \infty }\frac{1}{K}\sum_{k=0}^{K-1}X_{k}⩽\frac{b}{1-a}.$$

**Proof.**  From the sequence of upper bounds, we can inductively show that

$$X_{k}⩽a^{k}X_{0}+b\frac{1-a^{k}}{1-a},\;\forall \;k\in Z_{+}.$$

Averaging $X_{k}$ over the horizon {0,⋯,K−1}, we get

$$\frac{1}{K}\sum_{k=0}^{K-1}X_{k}⩽\frac{1-a^{K}}{K(1-a)}\left(X_{0}-\frac{b}{1-a}\right)+\frac{b}{1-a}.$$

From the finiteness of $X_{0},b$ and the fact that |a|<1, the result follows by taking the limit *K* growing arbitrarily large on both sides. □

We are now in a position to prove Theorem 3.

**Proof of Theorem 3.**  We begin by noting that, without any loss of generality, we can restrict to T=Ks. This holds since the contributions of the error term within the fixed interval $I_{K}$ are bounded. For T=Ks, the time duration {0,⋯,T} can be partitioned into intervals $\left(I_{k},k+1\in \left[K\right]\right)$. Therefore, we can write the average MSE per dimension for the *p*-SU scheme for time-horizon T=Ks as

$${\bar{D}}_{T}\left(\phi_{p},\psi_{p},X\right)=\frac{1}{Ks}\sum_{k=0}^{K-1}\sum_{j=0}^{m-1}\sum_{i=0}^{p-1}D_{ks+jp+i}\left(\phi_{p},\psi_{p},X\right).$$

From the upper bound for per dimension MSE given in Lemma 1, we get

$$\begin{array}{cc} \sum_{i=0}^{p-1}D_{ks+jp+i}\left(\phi_{p},\psi_{p},X\right) & ⩽\sum_{i=0}^{p-1}[\alpha^{2(jp+i)}\theta^{j}D_{ks}\left(\phi_{p},\psi_{p},X\right)+\sigma^{2}\left(1-\alpha^{2(jp+i)}\right)+\frac{\alpha^{2(jp+i)}\left(1-\theta^{j}\right)\varepsilon^{2}}{\left(1-\theta \right)}\\ & \;+\alpha^{2(jp+i)}\kappa \beta ]\\ & =\alpha^{2jp}\frac{1-\alpha^{2p}}{1-\alpha^{2}}\left(\theta^{j}D_{ks}\left(\phi_{p},\psi_{p},X\right)+\frac{\left(1-\theta^{j}\right)\varepsilon^{2}}{\left(1-\theta \right)}+\kappa \beta -\sigma^{2}\right)+p\sigma^{2}. \end{array}$$

Summing the expression above over j∈{0,…,m−1} and k∈{0,…,K−1}, and dividing by *T*, we get

$$\begin{array}{cc} {\bar{D}}_{T}\left(\phi_{p},\psi_{p},X\right)⩽ & \sigma^{2}+\frac{\left(1-\alpha^{2s}\right)}{s(1-\alpha^{2})}\left(\kappa \beta -\sigma^{2}+\frac{\varepsilon^{2}}{1-\theta }\right)\\ & +\frac{\left(1-\alpha^{2p}\right)}{s(1-\alpha^{2})}\frac{\left(1-\alpha^{2s}\theta^{m}\right)}{\left(1-\alpha^{2p}\theta \right)}\left(\frac{1}{K}\sum_{k=0}^{K-1}D_{ks}\left(\phi_{p},\psi_{p},X\right)-\frac{\varepsilon^{2}}{1-\theta }\right). \end{array}$$

It follows by Lemma 2 that

(5) $$\begin{array}{cc} {\bar{D}}_{T}\left(\phi_{p},\psi_{p},X\right)⩽ & \sigma^{2}+\frac{\left(1-\alpha^{2s}\right)}{s(1-\alpha^{2})}\left(\kappa \beta -\sigma^{2}+\frac{\varepsilon^{2}}{1-\theta }\right)\\ \; & +\frac{\left(1-\alpha^{2p}\right)}{s(1-\alpha^{2})}\frac{\left(1-\alpha^{2s}\theta^{m}\right)}{\left(1-\alpha^{2p}\theta \right)}\left(\underset{{\left\{a_{k}\right\}}_{k⩾0}\in A}{\sup}\frac{1}{K}\sum_{k=0}^{K-1}a_{k}-\frac{\varepsilon^{2}}{1-\theta }\right), \end{array}$$

where the A denotes the set of ${\left\{a_{k}\right\}}_{k⩾0}$ satisfying

$$\begin{array}{cc} a_{k} & ⩽\alpha^{2s}\theta^{m}a_{k-1}+\sigma^{2}\left(1-\alpha^{2s}\right)+\alpha^{2s}\kappa \beta +\alpha^{2s}\varepsilon^{2}\frac{\left(1-\theta^{m}\right)}{\left(1-\theta \right)}. \end{array}$$

We denote the right-side of (5) by $B_{T}\left(\theta ,\epsilon ,\beta \right)$. Noting that by Lemma 3 any sequence ${\left\{a_{k}\right\}}_{k⩾0}\in A$ satisfies

$$\begin{array}{c} \lim_{K\to \infty }\frac{1}{K}\sum_{k=0}^{K-1}a_{k}⩽\frac{1}{1-\alpha^{2s}\theta^{m}}\left(\sigma^{2}\left(1-\alpha^{2s}\right)+\alpha^{2s}\kappa \beta +\alpha^{2s}\varepsilon^{2}\frac{\left(1-\theta^{m}\right)}{\left(1-\theta \right)}\right), \end{array}$$

we get that

$$\begin{array}{cc} \underset{T\to \infty }{lim sup}B_{T}\left(\theta ,\epsilon ,\beta \right)⩽ & \sigma^{2}\left(1-\frac{\left(1-\alpha^{2s}\right)}{s(1-\alpha^{2})}\cdot \frac{\alpha^{2p}\left(1-\theta \right)}{\left(1-\alpha^{2p}\theta \right)}\right)+\varepsilon^{2}\frac{\left(1-\alpha^{2s}\right)}{s(1-\alpha^{2})}\cdot \frac{\alpha^{2p}}{\left(1-\alpha^{2p}\theta \right)}\\ & +\kappa \beta \frac{1}{s(1-\alpha^{2})}\left(1-\frac{\alpha^{2(s+p)}\left(1-\theta \right)}{\left(1-\alpha^{2p}\theta \right)}\right), \end{array}$$

which completes the proof. □

## 6. Asymptotic Achievability Using Random Quantizer

With Theorem 3 at our disposal, the proof of achievability can be completed by fixing p=1 and showing the existence of appropriate quantizer. However, we need to handle the failure event, and we address this first. The next result shows that the failure probability depends on the quantizer only through *M*.

**Lemma** **4.** *For fixed T and n, consider the p-SU scheme with p=1 and an nR bit (θ,ϵ)-quantizer Q with dynamic range M. Then, for every η>0, there exists an $M_{0}$ independent of n such that for all $M⩾M_{0}$, we get*

$$P\left(A_{T}^{c}\right)⩽\eta .$$

**Proof.**  The event $A_{T}$ (of encoder failure not happening until time *T* for the successive update scheme) occurs when the errors $Y_{k,j}$ satisfies ${\left\|Y_{k,j}\right\|}_{2}^{2}⩽nM^{2}$, for every k⩾0 and 0⩽j⩽s−1 such that t=ks+j⩽T. For brevity, we denote by $Y_{t}$ the error random variable $Y_{k,j}$ and $Y^{t-1}=\left(Y_{1},\ldots ,Y_{t-1}\right)$. We note that

$$\begin{array}{cc} P\left(A_{T}^{c}\right) & =P\left(A_{T-1}^{c}\right)+P\left(A_{T-1}\cap A_{T}^{c}\right)\\ & =P\left(A_{T-1}^{c}\right)+P\left(A_{T-1}\cap \left\{{\left\|Y_{T}\right\|}_{2}^{2}>nM^{2}\right\}\right)\\ & =P\left(A_{T-1}^{c}\right)+E\left[{𝟙}_{A_{T-1}}P\left({\left\|Y_{T}\right\|}_{2}^{2}>nM^{2}|Y^{T-1}\right)\right]\\ & ⩽P\left(A_{T-1}^{c}\right)+E\left[{𝟙}_{A_{T-1}}\frac{E[{\left\|Y_{T}\right\|}_{2}^{2}|Y^{T-1}]}{nM^{2}}\right]\\ & =P\left(A_{T-1}^{c}\right)+\frac{E[{\left\|Y_{T}\right\|}_{2}^{2}{𝟙}_{A_{T-1}}]}{nM^{2}}. \end{array}$$

Note that the inequality follows from Markovs inequality. Further we have $Y_{T}=Y_{T-1}-Q\left(Y_{T-1}\right)$ under $A_{T-1}$, whereby

$$E\left[{\left\|Y_{T}\right\|}_{2}^{2}{𝟙}_{A_{T-1}}\right]⩽\theta \cdot E\left[{\left\|Y_{T-1}\right\|}_{2}^{2}{𝟙}_{A_{T-1}}\right]+n\varepsilon^{2}.$$

Denoting by $\beta_{T}^{2}$ the probability $P\left(A_{T}^{c}\right)$, the previous two inequalities imply

$$\beta_{T}^{2}⩽\beta_{T-1}^{2}+\frac{\theta }{nM^{2}}E\left[{\left\|Y_{T-1}\right\|}_{2}^{2}\right]+\frac{\varepsilon^{2}}{M^{2}}.$$

We saw earlier in the proof of Lemma 1 that $E[{\left\|Y_{T-1}\right\|}_{2}^{2}]/n$ depends only on the probability $\beta_{T-1}^{2}$ that failure does not occur until time T−1. Proceeding as in that proof, we get

$$\beta_{T}^{2}⩽\beta_{T-1}^{2}+\frac{1}{M^{2}}\left(c_{1}\beta_{T-1}+c_{2}\right),$$

where $c_{1}$ and $c_{2}$ do not depend on *n*. Therefore, there exists $M_{0}$ independent of *n* such that for all *M* exceeding $M_{0}$ we have

$$\beta_{T}^{2}⩽\beta_{T-1}^{2}+\eta ,$$

which completes the proof by summing over *T*. □

The bound above is rather loose, but it suffices for our purpose. In particular, it says that we can choose *M* sufficiently large to make probability of failure until time *T* less than any $\beta^{2}$, whereby Theorem 3 can be applied by designing a quantizer for this *M*. Indeed, we can use the quantizer of unit sphere from [1,2], along with a uniform quantizer for gain (which lies in [−M,M]) to get the following performance. In fact, we will show that a deterministic quantizer with the desired performance exists. Note that we already considered such a quantizer in Example 3. However, the analysis there was slightly loose and it assumed the existence of an ideal shape quantizer.

**Lemma** **5.** *For every R,ε,γ,M>0, there exists an nR bit $\left(2^{-2(R-\gamma )},\varepsilon \right)$-quantizer with dynamic range M, for all n sufficiently large.*

**Proof.**  We first borrow a classic construction from [1,2], which gives us our desired shape quantizer. Denote by $S_{n}$ the (n−1)-dimensional unit sphere $\left\{y\in R^{n}:{\left\|y\right\|}_{2}=1\right\}$. For every γ>0 and *n* sufficiently large, it was shown in [1,2] that there exist $2^{nR}$ vectors C in $S_{n}$ such that for every $y\in S_{n}$ we can find $y^{\prime }\in C$ satisfying

$$\left\langle y,y^{\prime }\right\rangle ⩾\sqrt{1-2^{-2(R-\gamma )}}.$$

Denoting $\cos\theta =\sqrt{1-2^{-2(R-\gamma )}}$, consider the shape quantizer $Q_{R}\left(y\right)$ from [2] given by

$$\begin{array}{cc} Q_{R}\left(y\right) & ≜\cos\theta \cdot \underset{y^{\prime }\in C}{arg min}{\left\|y-y^{\prime }\right\|}_{2}^{2}\\ & =\cos\theta \cdot \arg\max_{y^{\prime }\in C}\left\langle y,y^{\prime }\right\rangle ,\;\forall \;y\in S_{n}. \end{array}$$

Note that we shrink the length of $y^{\prime }$ by a factor of cosθ, which will be seen to yield the gain over the analysis in Example 3.

We append to this shape quantizer the uniform gain quantizer $q_{M}:\left[0,M\right]\to \left[0,M\right]$, which quantizes the interval [0,M] uniformly into subintervals of length ϵ. Specifically, $q_{M}\left(a\right)=\varepsilon \left\lfloor a/\varepsilon \right\rfloor$ and the corresponding index is given by ⌊a/ε⌋. We represent this index using its ℓ≜⌈log(M/ε)⌉ bit binary representation and denote this mapping by $\phi^{M}:\left[0,M\right]\to {\left\{0,1\right\}}^{\ell }$.

For every $y\in R^{n}$ such that ${\left\|y\right\|}_{2}^{2}⩽nM^{2}$, we consider the quantizer

$$Q\left(y\right)=\sqrt{n}\cdot q_{M}\left(\frac{{\left\|y\right\|}_{2}}{\sqrt{n}}\right)\cdot Q_{R}\left(\frac{y}{{\left\|y\right\|}_{2}}\right).$$

For this quantizer, for every $y\in R^{n}$ with ${\left\|y\right\|}_{2}^{2}=nB^{2}$ such that B⩽M, we have

$$\begin{array}{cc} {\left\|y-Q(y)\right\|}_{2}^{2} & ={\left\|y\right\|}_{2}^{2}+{\left\|Q(y)\right\|}_{2}^{2}-2\left\langle y,Q(y)\right\rangle \\ & =nB^{2}+n{\hat{B}}^{2}\cos^{2}\theta -2nB\hat{B}\cos\theta \left\langle \tilde{y},Q_{R}\left(\tilde{y}\right)\right\rangle \\ & ⩽nB^{2}+n{\hat{B}}^{2}\cos^{2}\theta -2nB\hat{B}\cos^{2}\theta \\ & =nB^{2}\sin^{2}\theta +n{\left(B-\hat{B}\right)}^{2}\cos^{2}\theta \\ & ⩽nB^{2}\sin^{2}\theta +n\varepsilon^{2}\cos^{2}\theta \\ & ⩽nB^{2}2^{-2(R-\gamma )}+n\varepsilon^{2}, \end{array}$$

where the first inequality uses the covering property of C. Therefore, *Q* constitutes an nR+ℓ bit $2^{-2(R-\gamma ),\varepsilon }$-quantizer with dynamic range *M*, for all *n* sufficiently large. Note that this quantizer is a deterministic one. □

**Proof of Theorem 1.**  For any fixed β and ε, we can make the probability of failure until time *T* less than β by choosing *M* sufficiently large. Further, for any fixed R,γ>0, by Lemma 5, we can choose *n* sufficiently large to get an nR bit $\left(2^{-2(R-\gamma )},\varepsilon \right)$-quantizer for vectors *y* with ${\left\|y\right\|}_{2}^{2}⩽nM^{2}$. Therefore, by Theorem 3 applied for p=1, we get that

$$\begin{array}{c} \delta^{*}\left(R,s,X\right)⩾\frac{g\left(s\right)\;\alpha^{2}}{1-\alpha^{2}2^{-2(R-\gamma )}}\;\left(1-\frac{\varepsilon^{2}}{\sigma^{2}}-2^{-2(R-\gamma )}\right)-\frac{\kappa \beta g(s)}{\sigma^{2}\left(1-\alpha^{2s}\right)}\;\left(1-\alpha^{2(s+1)}\frac{1-\theta }{1-\alpha^{2}\theta }\right). \end{array}$$

The proof is completed upon taking the limits as ε,γ, and β go to 0. □

## 7. Converse Bound: Proof of Theorem 2

The proof is similar to the converse proof in [55], but now we need to handle the delay per transmission. We rely on the properties of *entropy power* of a random variable. Recall that for a continuous random variable *X* taking values in $R^{n}$, the entropy power of *X* is given by

$$N\left(X\right)=\frac{1}{2\pi e}\cdot 2^{\left(2/n)\;h(X\right)},$$

where h(X) is the differential entropy of *X*.

Consider a tracking code (ϕ,ψ) of rate *R* and sampling period *s* and a process $X\in X_{n}$. We begin by noting that the state at time *t* is related to the state at time t+i as

$$X_{t+i}=\alpha^{i}X_{t}+\sum_{j=0}^{i-1}\alpha^{j}\xi_{t+i-j},$$

where the noise $\sum_{j=0}^{i-1}\alpha^{j}\xi_{t+i-j}$ is independent of $X_{t}$ (and the past states). In particular, for t=ks+i, 1⩽i<s, we get

$$\begin{array}{cc} E\left[{\left\|X_{t}-{\hat{X}}_{t|t}\right\|}_{2}^{2}\right] & =E\left[{\left\|\alpha^{i}X_{ks}-{\hat{X}}_{t|t}\right\|}_{2}^{2}\right]+\sum_{j=0}^{i-1}\alpha^{2j}E\left[{\left\|\xi_{ks+i-j}\right\|}_{2}^{2}\right]\\ & =\alpha^{2i}E\left[{\left\|X_{ks}-{\tilde{X}}_{t}\right\|}_{2}^{2}\right]+n\left(1-\alpha^{2i}\right)\sigma^{2}. \end{array}$$

where we define ${\tilde{X}}_{t}:=\alpha^{-i}{\hat{X}}_{t|t}$ and the first identity uses the orthogonality of noise added in each round from the previous states and noise. The second equality follows directly by substituting the variance of the components of the process $\xi_{ks+i-j}$ and simplifying the summation. Since the Gaussian distribution has the maximum differential entropy among all continuous random variables with a given variance, and the entropy power for a Gaussian random variable equals its variance, we get that

$$\sigma^{2}\left(1-\alpha^{2}\right)⩾N\left(\xi_{t+i}\right).$$

Therefore, the previous bound for tracking error yields

(6) $$\begin{array}{cc} D_{ks+i}\left(\phi ,\psi ,X\right) & ⩾\alpha^{2i}\frac{1}{n}E\left[{\left\|X_{ks}-{\tilde{X}}_{ks+i}\right\|}_{2}^{2}\right]+\frac{\left(1-\alpha^{2i}\right)}{\left(1-\alpha^{2}\right)}N\left(\xi_{ks+i}\right)\\ & =\alpha^{2i}\frac{1}{n}E\left[{\left\|X_{ks}-{\tilde{X}}_{ks+i}\right\|}_{2}^{2}\right]+\frac{\left(1-\alpha^{2i}\right)}{\left(1-\alpha^{2}\right)}N\left(\xi_{1}\right), \end{array}$$

where the identity uses the assumption that $\xi_{t}$ are identically distributed for all *t*. Taking average of these terms for t=0,..,T, we get

$$\begin{array}{cc} {\bar{D}}_{T}\left(\phi ,\psi ,X\right) & =\frac{1}{nKs}\sum_{k=0}^{K-1}\sum_{i=ks}^{(k+1)s-1}E\left[{\left\|X_{i}-{\hat{X}}_{i|i}\right\|}_{2}^{2}\right]\\ & ⩾\frac{1}{nKs}\sum_{k=0}^{K-1}\sum_{i=0}^{s-1}\alpha^{2i}E\left[{\left\|X_{ks}-{\tilde{X}}_{ks+i}\right\|}_{2}^{2}\right]+\frac{N(\xi_{1})}{\left(1-\alpha^{2}\right)}\left(1-\frac{\left(1-\alpha^{2s}\right)}{s(1-\alpha^{2})}\right). \end{array}$$

Note that ${\tilde{X}}_{ks+i}$s act as estimates of $X_{ks}$ which depend on the communication received by the decoder until time ks+i. We denote the communication received at time *t* by $C_{t-1}$, whereby ${\tilde{X}}_{ks+i}$ depends only on $C_{1},\ldots ,C_{ks+i-1}$. In particular, the communication $C_{ks},\ldots ,C_{ks+i-1}$ was sent as a function of $X_{ks}$, the sample seen at time t=ks.

From here on, we proceed by invoking the “entropy power bounds” for the MSE terms. For random variables *X* and *Y* such that $P_{X|Y}$ has a conditional density, the conditional entropy power is given by $N\left(X|Y\right)=1/\left(2\pi e\right)2^{2h(X|Y)/n}$. (The conditional differential entropy h(X|Y) is given by $E\left[h(P_{X|Y})\right]$.) Bounding MSE terms by entropy power is a standard step that allows us to track reduction in error due to a fixed amount of communication.

We begin by using the following standard bound (see [56], Chapter 10): (It follows simply by noting that Gaussian maximizes differential entropy among all random variables with a given second moment and that h(X)−h(X|Y)⩽H(Y)=nR.) For a continuous random variable *X* and a discrete random variable *Y* taking ${\left\{0,1\right\}}^{nR}$ values, let $\hat{X}$ be any function of *Y*. Then, it holds that

(7) $$\frac{1}{n}E{\left\|X-\hat{X}\right\|}^{2}⩾2^{-2R}N\left(X\right).$$

We apply this result to $X_{ks}$ given $C^{ks-1}$ in the role of *X* and the communication $C_{ks},..,C_{ks+i-1}$ in the role of *Y*. The previous bound and Jensen’s inequality yield

$$\frac{1}{n}E\left[{\left\|X_{ks}-{\tilde{X}}_{ks+i}\right\|}_{2}^{2}\right]⩾2^{-2Ri}E\left[N\left(X_{ks}|C^{ks-1}\right)\right].$$

Next, we recall the entropy power inequality (cf. [56]): For independent $X_{1}$ and $X_{2}$, $N\left(X_{1}+X_{2}\right)⩾N\left(X_{1}\right)+N\left(X_{2}\right)$. Noting that $X_{ks}=\alpha^{s}X_{(k-1)s}+\sum_{j=0}^{s-1}\alpha^{j}\xi_{ks-j}$, where $\left\{\xi_{i}\right\}$ is an *iid* zero-mean random variable independent of $X_{(k-1)s}$, and that $C^{ks-1}$ is a function of $X_{1},\ldots ,X_{(k-1)s}$, we get

$$\begin{array}{cc} N(X_{ks}|C^{ks-1})\; & ⩾N\left(\alpha^{s}X_{(k-1)s}|C^{ks-1}\right)+N\left(\xi_{ks}\right)\frac{\left(1-\alpha^{2s}\right)}{\left(1-\alpha^{2}\right)}\\ \; & =\alpha^{2s}N\left(X_{(k-1)s}|C^{ks-1}\right)+N\left(\xi_{1}\right)\frac{\left(1-\alpha^{2s}\right)}{\left(1-\alpha^{2}\right)}, \end{array}$$

where the previous identities utilizes the scaling property of differential entropy. Upon combining the bounds given above and simplifying, we get

(8) $$\begin{array}{cc} {\bar{D}}_{T}\left(\phi ,\psi ,X\right) & ⩾\frac{\alpha^{2s}\left(1-\alpha^{2s}2^{-2Rs}\right)}{s(1-\alpha^{2}2^{-2R})}\cdot \frac{1}{K}\sum_{k=0}^{K-1}E\left[N\left(X_{(k-1)s}|C^{ks-1}\right)\right]\\ & \;+\frac{N(\xi_{1})}{\left(1-\alpha^{2}\right)}\left(1+\frac{\left(1-\alpha^{2s}\right)\left(1-\alpha^{2s}2^{-2Rs}\right)}{s(1-\alpha^{2}2^{-2R})}-\frac{\left(1-\alpha^{2s}\right)}{s(1-\alpha^{2})}\right). \end{array}$$

Finally, note that the terms $N(X_{(k-1)s}|C^{ks-1})$ are exactly the same as that considered in [55] (eqn. 11e) since they correspond to recovering $X_{(k-1)s}$ using communication that can depend on it. Therefore, a similar expression holds here, for the sampled process $\left\{X_{ks}:k\in N\right\}$. Using the recursive bound for the tracking error in (6) and (7), we adapt the results of [55] (eqn. 11) for our case to obtain

$$E\left[N\left(X_{(k-1)s}|C^{ks-1}\right)\right]⩾d_{k-1}^{*},$$

where the quantity $d_{k}^{*}$ is given by the recursion

$$d_{k}^{*}=2^{-2Rs}\left(\alpha^{2s}d_{k-1}^{*}+N\left(\xi_{1}\right)\frac{\left(1-\alpha^{2s}\right)}{\left(1-\alpha^{2}\right)}\right),$$

with $d_{0}^{*}=0$.

The bound obtain above holds for any given process $X\in X_{n}$. To obtain the best possible bound we substitute $\xi_{1}$ to be a Gaussian random variable, since that would maximize $N(\xi_{1})$. Specifically, we set $\left\{\xi_{k}\right\}$ to be a Gaussian random variable with zero mean and variance $\sigma^{2}$ to get $N\left(\xi \right)=\sigma^{2}\left(1-\alpha^{2}\right)$. Thus, taking supremum over all distributions on both sides of (8), we have

$$\begin{array}{cc} & \underset{X\in X_{n}}{\sup}{\bar{D}}_{T}\left(\phi ,\psi ,X\right)⩾\\ & \frac{\alpha^{2s}\left(1-\alpha^{2s}2^{-2Rs}\right)}{s(1-\alpha^{2}2^{-2R})}\cdot \frac{1}{K}\sum_{k=0}^{K-1}d_{k-1}^{*}+\sigma^{2}\left(1+\frac{\left(1-\alpha^{2s}\right)\left(1-\alpha^{2s}2^{-2Rs}\right)}{s(1-\alpha^{2}2^{-2R})}-\frac{\left(1-\alpha^{2s}\right)}{s(1-\alpha^{2})}\right), \end{array}$$

where

$$d_{k}^{*}=2^{-2Rs}\left(\alpha^{2s}d_{k-1}^{*}+\sigma^{2}\left(1-\alpha^{2s}\right)\right),$$

with $d_{0}^{*}=0$. For this sequence $d_{k}^{*}$, we can see that (cf. [55] (Corollary 1))

$$\begin{array}{c} \underset{K\to \infty }{lim sup}\frac{1}{K}\sum_{k=0}^{K-1}d_{k-1}^{*}=\lim_{K\to \infty }d_{k}^{*}=\frac{\sigma^{2}\left(1-\alpha^{2s}\right)2^{-2Rs}}{\left(1-\alpha^{2s}2^{-2Rs}\right)}. \end{array}$$

Therefore, we have obtained

$$\begin{array}{cc} \underset{T\to \infty }{lim sup}\underset{X\in X_{n}}{\sup}{\bar{D}}_{T}\left(\phi ,\psi ,X\right)\; & ⩾\sigma^{2}\left(\frac{\left(1-\alpha^{2s}\right)\alpha^{2s}2^{-2Rs}}{s(1-\alpha^{2}2^{-2R})}+1-\frac{\left(1-\alpha^{2s}\right)}{s(1-\alpha^{2})}+\frac{\left(1-\alpha^{2s}\right)\left(1-\alpha^{2s}2^{-2Rs}\right)}{s(1-\alpha^{2}2^{-2R})}\right)\\ \; & =\sigma^{2}\left(1-g\left(s\right)\delta_{0}\left(R\right)\right). \end{array}$$

As the bound obtained above holds for all tracking codes (ϕ,ψ), it follows that $\delta^{*}\left(R,s,X\right)⩽g\left(s\right)\delta_{0}\left(R\right).$

## 8. Discussion

We restricted our treatment to an AR[1] process with uncorrelated components. This restriction is for clarity of presentation, and some of the results can be extended to AR[1] processes with correlated components. In this case, the decoder will be replaced by a Kalman-like filter in the manner of [35]. A natural extension of this work is the study of an optimum transmission strategy for an AR[*n*] process in the given setting. In an AR[*n*] process, the strategy of refining the latest sample is clearly not sufficient as the value of the process at any time instant is dependent on the past *n* samples. If the sampling is periodic, even the encoder does not have access to all these *n* samples unless we take a sample at every instant. A viable alternative is to take *n* consecutive samples at every sampling instant. However, even with this structure on the sampling policy, it is not clear how the information must be transmitted. A systematic analysis of this problem is an interesting area of future research.

Another setting which is not discussed in the current work is where the transmissions are of nonuniform rates. Throughout our work, we have assumed periodic sampling and transmissions at a fixed rate. For the scheme presented in this paper, it is easy to see from our analysis that only the total number of bits transmitted in each sampling interval matters, when the dimension is sufficiently large. That is, for our scheme, even framing each packet (sent in each communication slot) using unequal number of bits will give the same performance as that for equal packet size, if the overall bit-budget per sampling period is fixed. A similar phenomenon was observed in [39], which allowed the extension of some of their analysis to erasure channels with feedback. We remark that a similar extension is possible for some of our results, too. This behavior stems from the use of successive batches of bits to successively refine the estimate of a single sample within any sampling interval, whereby at the end of the sampling interval the error corresponds to roughly that for a quantizer using the total number of bits sent during the interval. In general, a study of nonuniform rates for describing each sample, while keeping bits per time-slot fixed, will require us to move beyond uniform sampling. This, too, is an interesting research direction to pursue.

Finally, we remark that the encoder structure we have imposed, wherein the error in the estimate of the latest sample is refined at each instant, is optimal only asymptotically and is justified only heuristically for fixed dimensions. Even for one dimensional observation it is not clear if this structure is optimal. We believe that this is a question of fundamental interest which remains open.

## Data Availability

Not applicable.

## Abbreviations

The following abbreviations are used in this manuscript:

AR[1]	Auto-Regressive process of order 1
